# Supplementary figures and images for: The “DDVF” motif used by viral and bacterial proteins to hijack RSK kinases mimics a short linear motif (SLiM) found in proteins related to the RAS-ERK MAP kinase pathway
Source: PLoS Pathog. 2025 Mar 28;21(3):e1013016. doi: 10.1371/journal.ppat.1013016 (PMC11984722; doi:10.1371/journal.ppat.1013016)

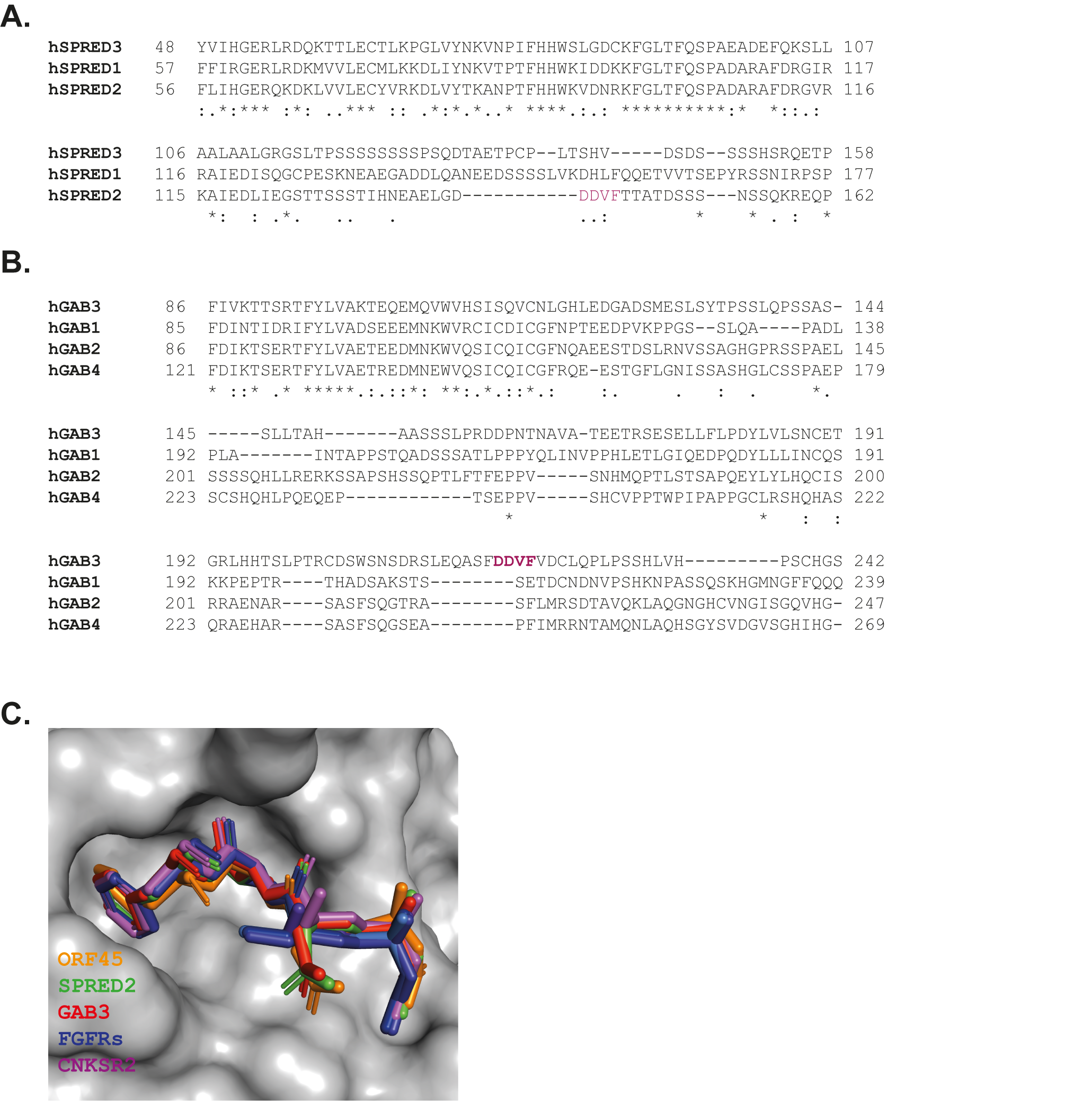

Supplement: S1 Fig — A-B. Conservation of DDVF (purple) motif across all three SPRED (A) and GAB (B) proteins. C. AlphaFold-multimer docks DDVF-like motifs from SPRED2 (green), GAB3 (red), FGFR1-4 (blue), and CNKSR2 (purple) in the RSK KAKLGM docking site. The predicted structures superimpose with that of the crystal structure of ORF45 DDVF (orange) bound to RSK2 (PDB 7OPO). (TIF) [file ppat.1013016.s004.tif]

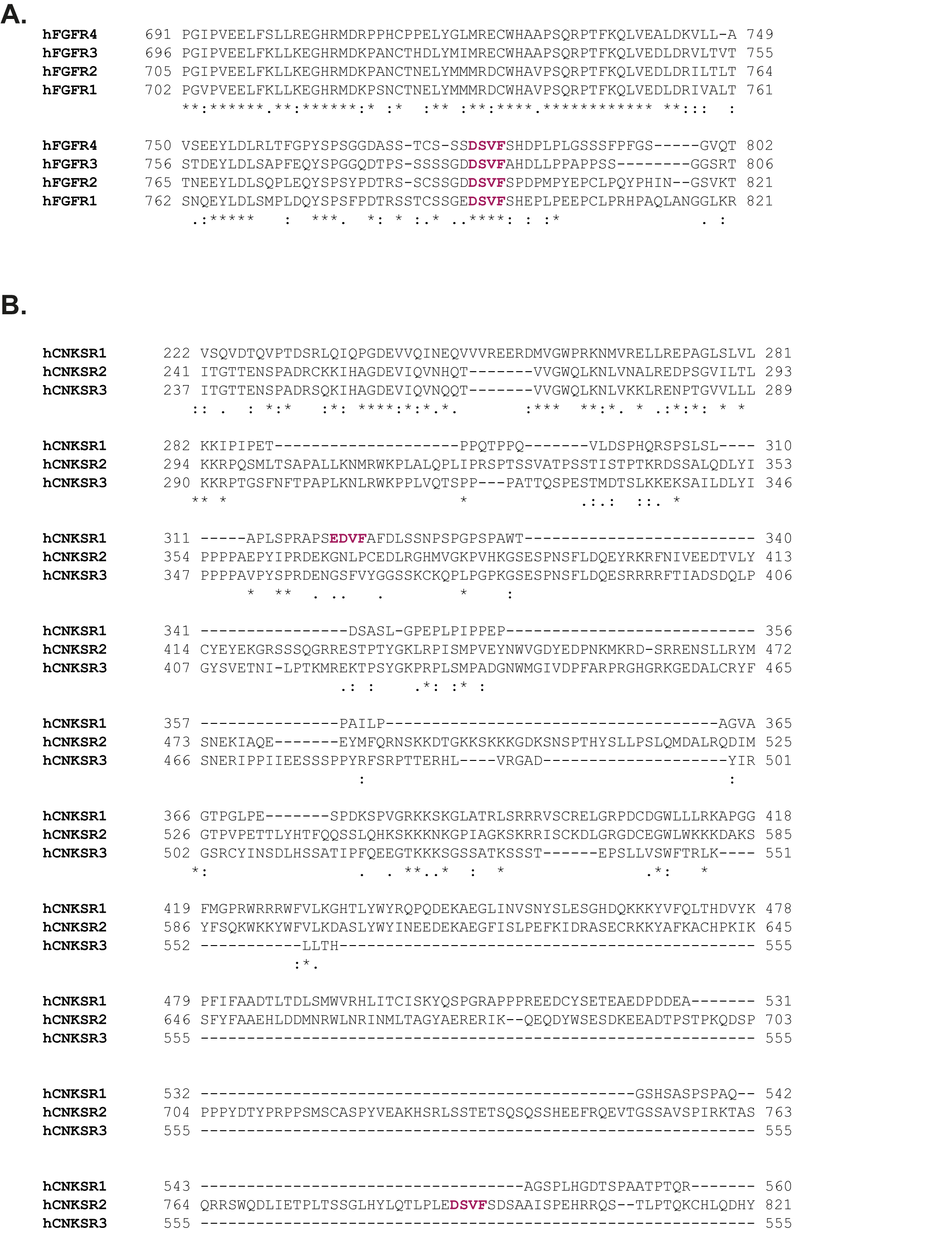

Supplement: S2 Fig — A-B. Conservation of the DSVF (purple) motif across all three FGFR (A) and CNKSR (B) isoforms. Note that CNKSR1 contains an EDVF motif which does not align with the DSVF motif of CNKSR2. (TIF) [file ppat.1013016.s005.tif]

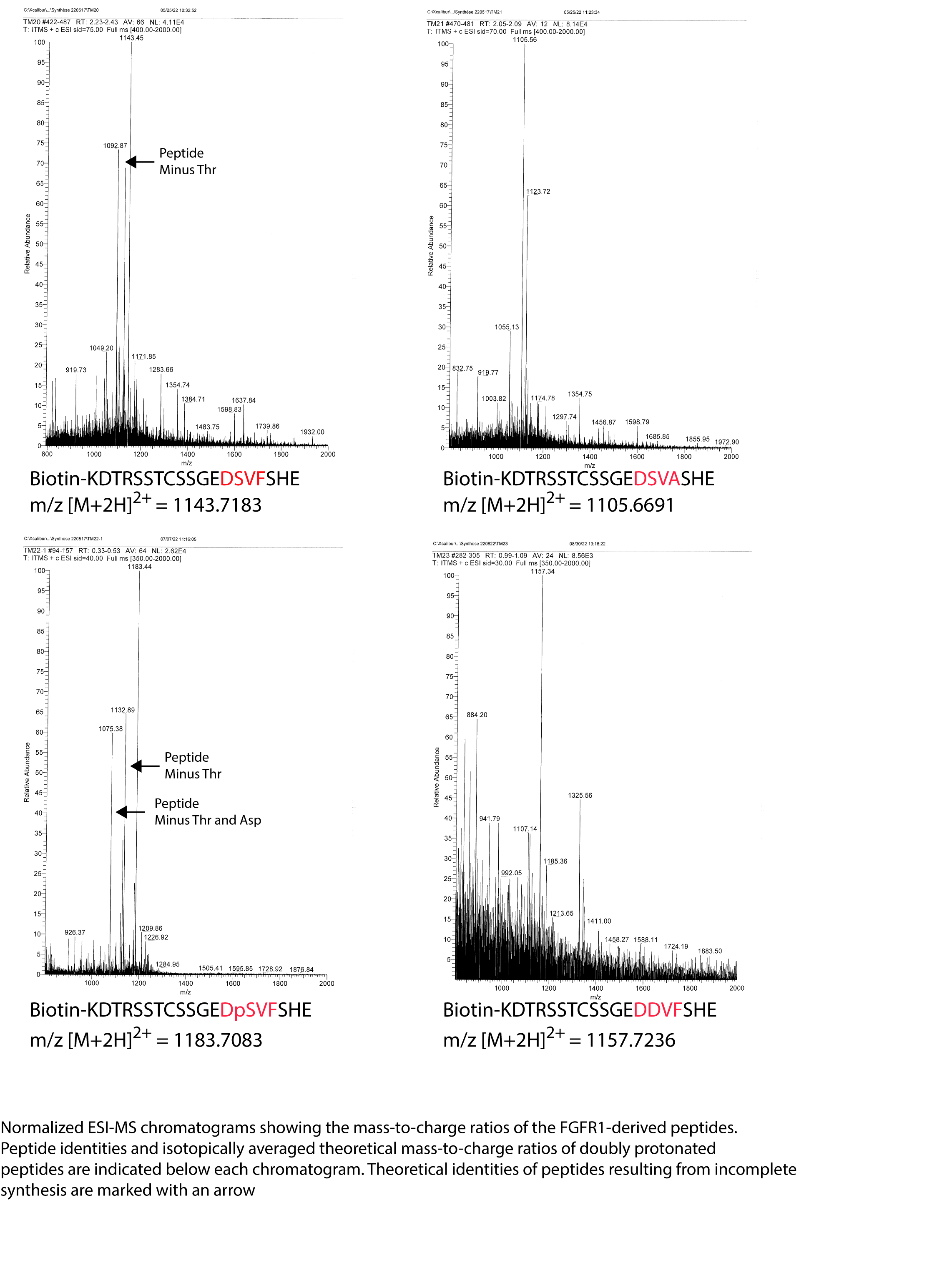

Supplement: S3 Fig — Peptide identities and isotopically averaged theoretical mass-to-charge ratios of doubly protonated peptides are indicated below each chromatogram. Theoretical identities of peptides resulting from incomplete synthesis are marked with an arrow. (TIF) [file ppat.1013016.s006.tif]

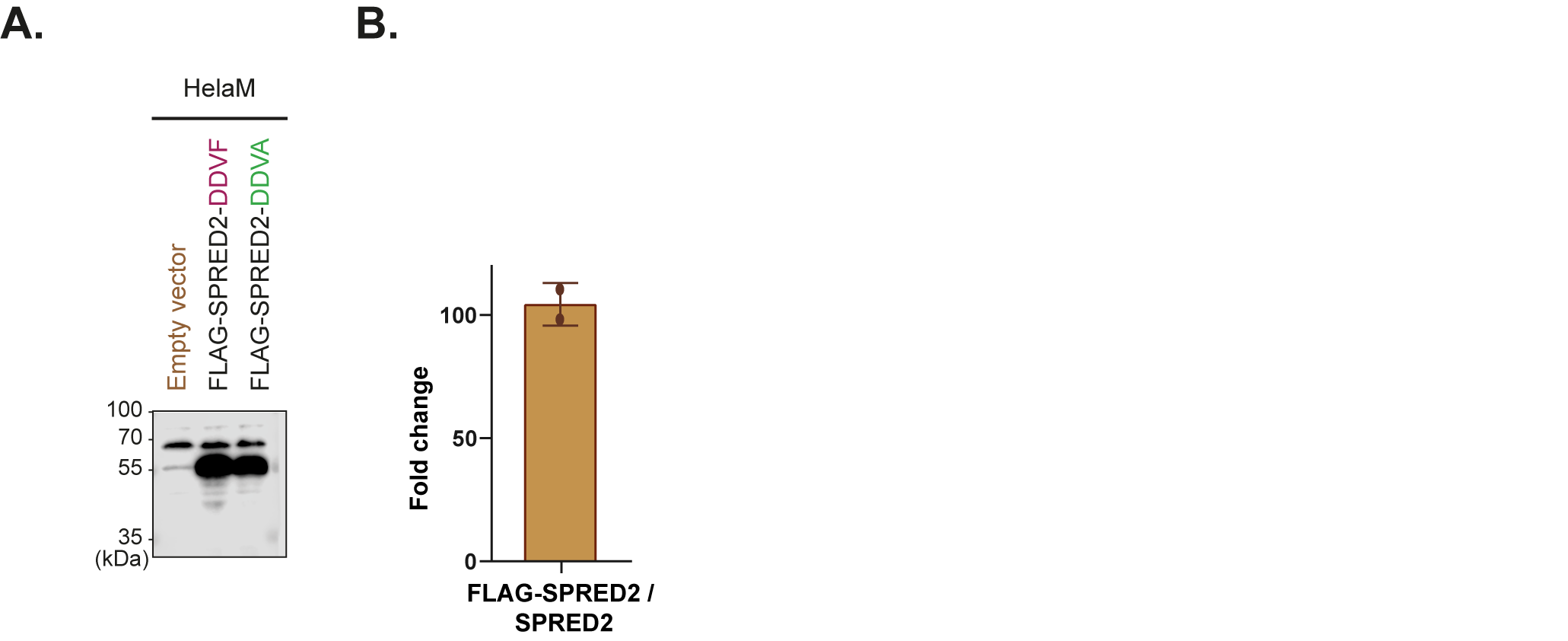

Supplement: S4 Fig — A. Immunoblot showing SPRED2 detection in HeLa M cells transduced with either an empty vector, FLAG-SPRED2 wild-type (WT), or the FLAG-SPRED2 DDVA mutant. B. Quantification of the immunoblot in panel A, displaying the calculated fold change between the levels of putative endogenous SPRED2 and the transduced FLAG-SPRED2. (TIF) [file ppat.1013016.s007.tif]
