# Supplementary material for: The “DDVF” motif used by viral and bacterial proteins to hijack RSK kinases mimics a short linear motif (SLiM) found in proteins related to the RAS-ERK MAP kinase pathway
Source: PLoS Pathog. 2025 Mar 28;21(3):e1013016. doi: 10.1371/journal.ppat.1013016 (PMC11984722; doi:10.1371/journal.ppat.1013016)
Supplement: Attachment — Submitted filename: Veinstein-Response to reviewers comments.pdf [file ppat.1013016.s008.pdf]

Reviewer #1: The authors examine Short Linear Motifs (SLiMs) that interact with RSKinases and are used by viruses to potentially dysregulate the RAS-ERK pathway. The authors use co-immunoprecipitation to identify two known and two novel cellular proteins that interact with RSKs using an interface found in proteins encoded by various pathogens. The authors also express a mutated form of ERK that lacks the docking site in HeLa cells, and results suggest that ERK may interact with multiple partners and be involved in a kind of negative feedback. Overall the results are clearly presented and convincing, although ultimately it remains unclear what exactly pathogens gain by modifying RSKs, other than general dysregulation of the RAK-ERK pathway, which is involved in cell cycle regulation and a wide range of other activities.

Reviewer #1: Are HeLa cells the best choice for studying RSK-mediated negative feedback? I understand that they are useful for many applications, but they are also heavily mutated and behave irregularly, in particular with regards to cell cycle control. Would it be worth providing some discussion of this in the text to put it into context? The results seem logical, but it is unclear to me how much they can be extended to real-world infection dynamics.

> We thank this reviewer for his positive comments and agree on the fact that HeLa cells may not be the best choice for studying cell cycle-related signaling. We were however limited by the availability of biological tools, including RSK-KO cells. Specifically, studying RSK-mediated feedback requires reducing the expression of all four RSK isoforms (RSK1-4), as they share the same KAKLGM site, a process that is both time-intensive and technically demanding. Cells also needed to be easy to transfect, to transduce, etc.
To extend the results to "infection dynamics", we added an experiment (Fig 7) where L929 cells were infected with Theiler's virus derivatives expressing either the wild type L protein or L mutants affected in their ability to bind RSK or to cause nucleo-cytoplasmic trafficking perturbation. Results of this experiment confirm that the DDVF motif of L plays a dual role as it allows both hijacking RSKs to trigger nucleo-cytoplasmic trafficking perturbation and competition with endogenous protein leading to ERK signaling dysregulation.

Reviewer #1: Line 420 – Clustal Omega likely works fine for these applications, and in many cases it is possible to manually curate the short alignments anyway, but in the future the authors may wish to consider using the newest Muscle5 algorithm, which substantially outperforms clustal omega.

> We thank the reviewer for this suggestion. In this study, we used Clustal Omega as our standard multiple sequence alignment algorithm due to its ease of use and broad online accessibility. For future analyses, we will consider using Muscle5.

For AlphaFold multimer results, what are the pLDDT values? I am curious to know if the authors had a cutoff that they used for these, or how they assessed the quality of the structural predictions. I understand that some of these results are available in the Zenodo repository provided, but given the importance of these values for assessing the results I believe they should be reported in the text.

> To clarify this point, we did not use pLDDT values to assess the binding of protein candidates to RSK. One reason for this is that, in some cases, the pLDDT values for the “DDVF” regions in non-binding proteins were higher than those observed in actual RSK-binding proteins. Instead, we evaluated each AlphaFold prediction model by measuring the distance between the Cε2 of the phenylalanine in the DDVF motif and the F303 Cδ2 of RSK. These distances were then compared with those observed in the crystal structure of the ORF45:RSK complex (PDB:7OPO). A distance of less than 5 Å between the F Cε2 of DDVF motif and the F303 Cδ2 of RSK was considered indicative of an interaction. To improve clarity, these measurement data have been incorporated into Table S1A.

Reviewer #2: In this manuscript, Veinstein and colleagues ...
Aside from one figure showing changes in phosphorylation of ERK and RSK, it's also unclear whether these interactions between RSK and DDVF-containing proteins has a meaningful impact on MAPK signaling and cell behavior.

MAJOR Reviewer #2: The most conspicuous gap in this study is that the authors have not experimentally addressed the layer of regulation by pathogens. If these pathways and interactions are exploited by viruses and bacteria, it would be important to demonstrate that introduction of at least one protein from a pathogen interferes with the feedback loop on RSK – after all, the PLOS Pathogens audience is interested in pathogens.
Citation of previous work demonstrating how viral and bacterial proteins hijack the RSK family through SLiM mimicry is insufficient because readers will need to appreciate the extent to which this pathway and the interactions between the proteins described in this manuscript can be manipulated during infection (for example, by Kaposi sarcoma-associated herpes virus protein ORF45).

> We agree that the absence of direct evidence of pathogen interference with ERK signaling through DDVF competition was a limitation in our original manuscript. To address this, we conducted additional experiments to investigate how pathogen-derived DDVF-containing proteins compete with endogenous proteins for RSK binding and used Theiler's virus infection to assess DDVF-dependent ERK signaling perturbation in infected cells (see below).

The authors should:

1. Perform competition assays to see if a pathogen-associated protein can block the interaction between RSK and DDVF-containing proteins (such as with SPRED2 and GAB3 in Fig 1).

> Competition experiments were performed, between YopM and SPRED2 in a transfection setting (Fig 5) as well as between L and SPRED2 in an infection setting (Fig 6).

2. Perform a signaling experiment as in Fig 4D-E that includes a pathogen-associated protein as a comparison. For example, wild type cells should be compared head-to-head with cells expressing ORF45, and directly compared to the RSK knockout, RSK-KAKLGM, and RSK-KSEPPY.

> This experiment was tested with YopM but halted due to the difficulty to keep stable and even expression of the various proteins involved. Also, YopM or L were preferred to ORF45 because ORF45 was reported to bind to ERK as well as to RSK (Alexa et al., Nature Communications, https://doi.org/10.1038/s41467-022-28109-x), making the interpretation of the results more difficult. Instead, we analyzed, in infected cells, how the DDVF motif of Theiler's virus L affected ERK signaling (see point 3 below).

3. It would also be helpful if the authors could demonstrate that interrupting these DDVF-RSK interactions has a measurable effect on some aspect of cell behavior (downstream of pERK). Does this actually impact cell survival, growth, and translation to the benefit of a virus or bacteria?

> We analyzed ERK and RSK phosphorylation, as well as phosphorylation of eIF4B (an RSK substrate) in cells infected with Theiler's viruses that expressed either, L^{WT} which interacts with RSK but also triggers nucleo-cytoplasmic trafficking disruption, L^{F48A} which fails to interact with RSK (and thereby lost the ability to disrupt the nucleo-cytoplasmic traffic) or L^{M60V} which does interact with RSK but does not interfere with nucleo-cytoplasmic trafficking.
Results of this experiment (Fig. 7) show that L expression affects ERK signaling in a DDVF-dependent manner but that RSK downstream events are even more affected by nucleo-cytoplasmic disruption.

Minor Reviewer #2: 1. Lines 106-108 are not easy to understand. Could the authors please rephrase to be clearer?

> We modified the sentence to make it clearer (revised lines 114-118).

2. PMA treatment in Figure 2 was not explained in the text. Why was PMA used? What were the authors expecting? Is the absence of any obvious change in the pulldowns +/ PMA surprising?

> We thank the reviewer for pointing out this omission. We indeed performed co-immunoprecipitation experiments to assess the interaction between RSK1 and the FGFR1 receptor in both the absence and presence of PMA. This approach was informed by the findings of Nadratowska-Wesolowska and colleagues (Oncogene, 2014; 33(40):4823-36), who showed that the interaction between the C-terminal tail of FGFR1 and RSK2 increased upon stimulation with FGF1, EGF, or serum. They further demonstrated that this interaction diminished following pretreatment with PD173074, a specific FGFR inhibitor, or U0126, a MEK kinase inhibitor. Based on these findings, we hypothesized that activation of the ERK-MAPK pathway could facilitate the detection of an interaction between RSK1 and FGFR1, which justified our use of PMA stimulation in this context. However, in our experiments, pretreating cells with PMA prior to co-immunoprecipitation resulted in little to no detectable increase in the association of RSK1 with FGFR1.
We included an explanation in the revised manuscript (see lines 174-177)

3. Phosphorylation of FGF1R peptide increasing binding by only 25% (and it's not particularly obvious in Fig 2C). Is this difference in binding physiologically relevant? Could the authors please discuss this and comment on whether this seemingly small effect is meaningful in the context of RAS-MAPK activation?

> We do not know the physiological relevance of this observation. In both experiments (Figs 2C and 2D) the difference of binding between phospho- and non-phospho-peptides was however reproducible. We modified a sentence in the discussion to acknowledge that the effect was quite modest (rev lines 399-400) and the relevance incertain.

4. With the model figure in Fig 4A, it would be helpful if the authors could somehow denote previously known interactions and/or label the new biological relationships described in this manuscript. It's unclear from the figure whether any of these interactions and feedback mechanisms are supported by data from this manuscript (other than the purple shading of proteins with DDVF motifs).

We thank the reviewer for raising this point. We have updated the figure to clearly distinguish previously known RSK interactions (represented by black arrows in Fig 4A) from newly identified ones (grey arrows). To further clarify this point, we modified the legend of Figure 4A with a short explanation and reference to reported observations concerning those proteins.

5. It remains difficult to discern how much relative influence the proteins with DDVF motifs have on the negative regulation of RSK. For example, the KAKLGM-to-KSEPPY mutation reduces the inhibitory capacity of RSK on the pERK/ERK ratio by roughly 2- to 3-fold at the 15 min time point (Fig 4E, top panel). How does this level of pERK/ERK ratio compare to the same experiment (stimulation with bFGF) if done in a SPRED2 knockout? A GAB3 knockout? A CNKSR2 knockout? If you observe more activation in knockout cells than with the KAKLGM-to-KSEPPY RSK mutation, that would tell us that there are likely other domains and/or unknown interactions with these proteins that are more important regulating the RAS-MAPK pathway than the DDVF-KAKLGM interaction. Alternatively, if you see the same level of activation in the knockouts (and no further increase in pERK/ERK with the KAKLGM-to-KSEPPY mutant) then it would suggest that most or all regulation is occurring through the DDVF-KAKLGM interaction, which would be an important result.

> We agree that digging deeper into ERK regulation through the DDVF-KAKLGM interaction would be of interest. However, given the multiplicity of actors/interactors in this pathway, some of which

likely still to be identified, we are afraid that this analysis would be beyond the scope of this manuscript.

Reviewer #3: In the manuscript "The 'DDVF' motif used by viral and bacterial proteins to hijack RSK kinases evolved as a mimic of a short linear motif (SLiM) found in proteins related to the RAS-ERK MAP kinase pathway.", Veinstein et al. demonstrate a strategy to use pathogen-encoded motifs to identify new, host-host protein interactions using an AlphaFold-based model and then validating with pulldown assays. They also demonstrate that the DDVF motif is interchangeable with a DpSVF motif that the authors hypothesize may be used to regulate interactions with (some?) host binding partners. While similar to other alphafold guided techniques, the use of a pathogen protein as "bait" is an interesting approach, and potentially identifies an important area of host-pathogen interaction regulating the cell cycle. However, this study seems premature, as they do not demonstrate whether the pathogen encoded SLiMs they previously identified can compete with the native motifs to regulate the ERK-MAPK pathway, or that these phosphorylation events do regulate downstream events in the host relevant to host-pathogen interactions.

MAJOR Reviewer #3:

1. The authors make the claim in several locations (eg lines 40-42 and in discussion) "[Previously described pathogen motif bearing proteins] …also likely compete with human proteins". However, they do not provide any experiments to test this hypothesis. It is important to determine whether the pathogen-encoded DDVF SLiMs do in fact inhibit, modulate, or otherwise interfere with host DDVF-KAKLGM interactions, especially for a pathogen-focused journal. Similarly, some of the previously described pathogen-encoded proteins should be included as controls, eg in Fig. 1 one or more pathogen SLiM-encoding protein should be included as a control to determine whether there may be any difference in binding affinity, etc between host and pathogen proteins.

> We agree with this Reviewer (and the other Reviewers) that a limitation of our original manuscript was the lack of evidence that DDVF-containing pathogens' altered ERK signaling. As stated above in response to Rev #1, we performed additional experiments to document competition between pathogens' proteins and endogenous DDVF-containing proteins for RSK binding (in a transfection setting - Fig 5) as well as in an infection setting (Fig 6). Moreover, we further showed that infection with Theiler's virus affects ERK signaling in a DDVF-dependent fashion (Fig 7).

2. Fig. 2C (Line 179): If purity is an issue with these peptides, then this needs to be discussed. As stated in their manuscript, it's unclear what the contaminants are, and as such is difficult to assess these blots and their accuracy. Is this non-specific binding, or is this intended to reflect incomplete phosphorylation of the synthetic peptide? In which case how much of the peptide was phosphorylated? Similarly, Fig 2D. "Routine mass spectrometry" is mentioned and the data should be included.

> As recommended by this Reviewer, we added the data as a supplemental Fig S3. Also, in response to a request by Reviewer #2, we modified a sentence in the discussion to acknowledge that, although the findings were reproducible, their physiological significance was uncertain. In spite of those limitations, the fact that phosphatase treatment of the phospho-DpSVF phosphopeptide (but not of the non-phospho DSVF peptide) decreased binding strongly supports the influence of phosphorylation on binding.

3. Fig. 4: This is unconvincing for the role of the DDVF SLiM. The cellular model tests KAKLGM, which while the authors have shown this interacts with (some) DDVF SLiM proteins, they do not show the inverse, that only DDVF proteins interact with this KAKLGM, or that it is the DDVF-KAKLGM interaction mediating the phenotype they report. The authors also state that the SLiM binding site regulates RSKs' physiological activity, while only the phosphorylation of RSK and ERK were measured, lacking information on downstream RSK substrates.

> Given the difficulty to show that only DDVF proteins interact with the KAKLGM motif of RSK, we tried to strengthen our observations by analyzing ERK and RSK activation as well as phosphorylation of the downstream RSK target eIF4B in an infection setting (Fig 7)(Also in response to Rev #2). In this experiment, cells were infected with Theiler's virus derivatives that express either, L^{WT} which interacts with RSK but also triggers nucleo-cytoplasmic trafficking disruption, L^{F48A} which fails to interact with RSK (and thereby lost the ability to disrupt the nucleo-cytoplasmic traffic) or L^{M60V} which does interact with RSK but does not interfere with nucleo-cytoplasmic trafficking. Results of this experiment (Fig. 7) show that L expression affects ERK signaling (phospho-ERK as well as

phospho-RSK) in a DDVF-dependent manner (see also revised discussion lines 420-424). RSK downstream events (phospho-eIF4B) are however more affected by L protein-induced nucleo-cytoplasmic disruption than by ERK signaling (see lines 340-343).

Minor: Reviewer #3:

1. Fig. 1C and 1D: It is inaccurate to claim that RSK1 didn't IP with SPRED1 as a band can be seen on the figure. SPRED2 is substantially higher, but SPRED1 still IPs better than the SPRED2 mutant or RSK1 mutant. The GAB3 mutant also IPs with RISK1.

> We have updated the text to address this point and be more accurate. See for instance rev lines 133-136.

2. The authors state in multiple places that pathogens evolved to mimic this human motif, but evolutionary analysis is limited to alignments suggesting the motif is well conserved. There's no evolutionary analysis to support the claim that pathogens acquired this motif via mimicry evolution. The authors should soften their conclusions, as this is a difficult conclusion to demonstrate. …

>We agree with the reviewer's observation and have revised the text to soften our conclusions. This includes modifying the title to: *"The "DDVF" motif used by viral and bacterial proteins to hijack RSK kinases ~~evolved as a~~ mimics ~~of~~ a short linear motif (SLiM) found in proteins related to the RAS-ERK MAP kinase pathway."* The body of the text has been modified accordingly.

… Similarly, a discussion of why different species were chosen in these alignments for Figs 1A, 1B, 2A and 3A. If some of these genes aren't encoded by the same set of species, that is relevant information about how conserved these motifs may be.

> We agree with the reviewer on the importance of maintaining consistency in species selection across the alignment figures. To address this, we standardized the species used, ensuring they are representative of vertebrates to better highlight the conservation of the DDVF-like motif. Specifically, we selected species with well-annotated genomes and clearly identified homologs in the NCBI Gene database. Compared to the original Figures 1–3, Drosophila melanogaster and Anopheles gambiae were excluded, as homologs of GAB3 and FGFR1 could not be confidently identified in these invertebrates. Alignments in Figures 1A, 1B, 2A, and 3A now feature the same set of species for consistency.

3. Full-length alignments of FGFR isoforms and CNKSR isoforms should be included in the supplemental figures as it was done with SPRED and GAB.

> As for SPRED and GAB isoforms, we added extended alignments of FGFR(C-term) and CNKSR isoforms in the supplemental figures S2A-B. We briefly discuss those in the text.

4. Fig. 2B: The purpose and the result of the PMA induction were never discussed in the paper.

> Thank you for raising this point which was also raised by Reviewer#2. We indeed performed co-immunoprecipitation experiments to assess the interaction between RSK1 and the FGFR1 receptor in both the absence and presence of PMA. This approach was informed by the findings of Nadratowska-Wesolowska and colleagues (Oncogene, 2014; 33(40):4823-36), who showed that the interaction between the C-terminal tail of FGFR1 and RSK2 increased upon stimulation with FGF1, EGF, or serum. They further demonstrated that this interaction diminished following pretreatment with PD173074, a specific FGFR inhibitor, or U0126, a MEK kinase inhibitor. Based on these findings, we hypothesized that activation of the ERK-MAPK pathway could facilitate the detection of an interaction between RSK1 and FGFR1, which justified our use of PMA stimulation in this context. However, in our experiments, pretreating cells with PMA prior to co-immunoprecipitation resulted in little to no detectable increase in the association of RSK1 with FGFR1.
We included an explanation in the revised manuscript (see lines 174-177)

5. Line 232: Need to define bFGF.
> This was done. Thank you.

6. Table S1: summary - the description of column “AFm_Dock” is the same as “DDVF dock”.

> We replaced “DDVF dock” by “AFm_Dock” to remove this redundancy.

Table S1B doesn’t have AFm_Dock data.

> Given the time need for AlphaFold multimer predictions on our cluster, we could not do the additional 1302 predictions in Table S1B in a reasonable time.

The information in S1C and S1D appears to have already been included in S1A and S1B, respectively.

> Table S1C and S1D were merged to S1A and S1B respectively.

7. Line 287: Typo. CNKSR2 contains a DSVF motif.

> This was corrected. Thank you.
